# Whatever you want: Inconsistent results are the rule, not the exception, in the study of primate brain evolution

**DOI:** 10.1371/journal.pone.0218655

**Published:** 2019-07-22

**Authors:** Andreas Wartel, Patrik Lindenfors, Johan Lind

**Affiliations:** 1 Centre for Cultural Evolution and Department of Zoology Stockholm University, Stockholm, Sweden; 2 Institute for Future Studies, Stockholm, Sweden; Pavol Jozef Safarik University in Kosice, SLOVAKIA

## Abstract

Primate brains differ in size and architecture. Hypotheses to explain this variation are numerous and many tests have been carried out. However, after body size has been accounted for there is little left to explain. The proposed explanatory variables for the residual variation are many and covary, both with each other and with body size. Further, the data sets used in analyses have been small, especially in light of the many proposed predictors. Here we report the complete list of models that results from exhaustively combining six commonly used predictors of brain and neocortex size. This provides an overview of how the output from standard statistical analyses changes when the inclusion of different predictors is altered. By using both the most commonly tested brain data set and the inclusion of new data we show that the choice of included variables fundamentally changes the conclusions as to what drives primate brain evolution. Our analyses thus reveal why studies have had troubles replicating earlier results and instead have come to such different conclusions. Although our results are somewhat disheartening, they highlight the importance of scientific rigor when trying to answer difficult questions. It is our position that there is currently no empirical justification to highlight any particular hypotheses, of those adaptive hypotheses we have examined here, as the main determinant of primate brain evolution.

## Introduction

The field of primate brain evolution can be characterized as an array of contradicting results [1, 2]. Probably the most frequently phylogenetic comparative method used is phylogenetic generalized least squares regression (PGLS). Brain or neocortex size have often been the dependent variables, in combination with a varying number of predictor variables, depending on the hypothesis at hand. As conflicting results abound, an evaluation of this approach has its merits. Therefore, we here systematically vary choice of data set and inclusion/exclusion of predictor variables in the PGLS framework, to investigate if, why, and when contradictory results emerge.

Most previous studies have relied on one of only two available datasets on brain size. These datasets have been obtained using different methods—(i) by the estimation of weight for different brain parts of fresh brains [3] and (ii) the estimation of endocranial volume as a proxy for brain size [4]. Due to this difference, the two datasets are not appropriate to pool in statistical analyses. Here, we include new data [5], added to one of the old datasets [3], to broaden the reanalysis.

Though initially eager to reach interesting biological conclusions from the new data, this paper is foremost focused on evaluating the validity of previous analyses on brain size evolution in nonhuman primates. Our choices of both method and data are therefore based on what is praxis in the field of primate brain evolution. This study is not driven by any particular biological hypothesis and seeks only to reach conclusions on the reliability of previous results.

There exist many suggested non-mutually exclusive hypotheses for causes of variation in size and architecture of primate brains. Here, we summarize seven such particularly popular hypotheses that have been alternately supported and rejected in various studies.

### Allometric relationships

Brains are similar to other organs in that they scale allometrically with body size. Similarly, brain parts in turn scale allometrically with brain size. Simply put, larger brains are required to run larger bodies. Most differences in brain size and brain architecture between species can thus be predicted by body size [6, 7]. Due to such known allometric relationships, one usually controls for body size / brain size in evolutionary studies of primate brains. Whatever residual variation is left is the target for tests of adaptive hypotheses. The rationale here is that “intelligence” corresponds to the amount of excess brain mass after controlling for brain mass dedicated to bodily functions [8–11]. However, body size alone accounts for more than 90% of the variation in brain size differences between primates [8, 12](see also Results in this study), so only little is left to explain.

### General cognitive abilities

Larger relative brain size or brain component size has evolved to meet higher cognitive demands [8, 13–18].

### The social brain hypothesis

Some primates evolved large brains and/or larger brain components (neocortex) as social complexity is hypothesized to be more cognitively demanding [19–25].

### Sexual selection

Demands of sociality are different between males and females. This should produce detectable differences in relative brain size or brain component size between species where sexual selection is high compared to species where it is relaxed [10, 26–30].

### Diet

Fruits are harder to find than leaves, so some primates have evolved larger brains and/or large specific brain parts to cope with “a challenging diet of fruit” [31–35]([31] p. 312). Alternatively, the causal relationship is hypothesized to go in the other direction, that larger brains (that evolved for another reason) demands a more high-calorie diet [36, 37].

### Life history

Variation in juvenile period and life span is hypothesized to affect brain size evolution [38, 39]. An extended juvenile learning is necessary to evolve a bigger brain [40, 41]. Also, a longer life span is a consequence of slow growth in order to cope with the high energy costs of developing a large brain [42–44] and/or to facilitate more opportunities to harness the products of enhanced brain size [38, 45].

### The mosaic brain hypothesis

This is a composite hypothesis where it is hypothesized that “variation in the size of individual brain components reflects adaptive divergence in brain function mediated by selection” [46], p. 2, [27, 47, 48]. Here, all hypotheses can come into play simultaneously [21].

The list of competing hypotheses can go on [1, 2, 38] and as we have focused on the most common, there are some that we have left out. But the message from the literature is clear: there is no real consensus about the adaptive explanations for neither primate brain size nor primate brain architecture, or alternatively, the number of factors in play when it comes to brain evolution is huge. Many studies have sought to identify the main evolutionary drivers of primate brain evolution, where residual brain size or different aspects of brain architecture have been used as approximations of intelligence, making it difficult and unjustified to highlight any particular hypothesis from the smorgasbord of published significant results.

Because results have proven both ambiguous and contradictory, we use new data [5] in combination with a classic brain data set [3] and report the complete list of models that results from exhaustively combining six commonly used predictors (female group size, male group size, female sexual maturity, life span, innovation, and percent fruit in diet). Our choice of predictors in this study reflect our notion of what hypotheses are common. We do not attempt an exhaustive combination of all predictors that have been proposed in the literature as this would just add to the point we can already make from the variables included. Others have used combinations of other predictors than those we use here [39, 49].

We start out by calculating the ‘best’ model according to the Akaike information criteria (AIC) both when using total brain size as the dependent variable and when using neocortex size as the dependent variable. Then we use this output and examine the stability of results when the inclusion of different predictors is altered. We end by examining the stability of previously published analyses in the same way.

Our aim here is not to reach a final verdict on the biological relevance of different hypotheses, but to investigate if data and methods currently at hand are productive enough for such considerations at all. As our results indicate, the number of hypothetical causes of primate brain evolution is currently too large in relation to the number of data points for any clarifying analyses to be possible.

## Method

All data used in this study were collected from published literature and are presented in supporting information (S1 Table). Most studies on primate brain evolution have relied on Stephan et al.‘s classic data set on primate brains [3], for example [14, 19, 20, 25–27, 33, 34, 37, 38, 40, 41, 43, 46, 49–66]. We pool this dataset with new data [5] by calculating weighted averages.

Life history and body size data were obtained by a similar pooling procedure of two datasets [35, 67]. Length of juvenile period is approximated by age of sexual maturity. Life span is calculated as the period between sexual maturity and maximum recorded age at death. Percentage fruit in diet were obtained by pooling data from several sources [35, 51, 67, 68]. Rates of innovation were gathered from [14].

We used female weight as a proxy for species weight as it is less variable than male weight among species, as variation in male weight in sexually selected species to a large degree is a consequence of selection on physical strength [69].

Though there are several ways to quantify social complexity, e.g. pair-bonding [20], tactical deception [25], we use group size as it is the most common used approximation of social complexity (e.g. [26, 27, 32, 40, 41, 53, 56, 70]). In this study both male and female group sizes are used because it has previously been shown that female rather than male group size correlate positively with neocortex volume in primates [26, 27], suggesting that it is social demands on females that mainly drives primate brain evolution.

Number of species with full data on all variables (including phylogeny) and thus included in all analyses are *N* = 40.

### Statistical analysis

All analyses were executed in R [71] using the packages NLME [72], APE [73], MASS [74] and BRMS [75]. All variables were log-transformed prior to analysis except percentage fruit in diet, which instead were arcsine-square root transformed.

We used phylogenetic generalized least squares (PGLS) regressions throughout. This method allows for the estimation of the impact of phylogeny on the covariance among residuals, thereby controlling for relatedness [76, 77]. A consensus phylogeny for each dataset were obtained from [78]. Lambda λ was estimated but in some cases when lambda is very close to 1, processing in R sometimes crash due to an optimization error. When this happened lambda was fixed to 1 [78].

All combinations of the following variables were used as predictors: female group size, male group size, female sexual maturity, life span, innovation, and percent fruit in diet, both when using total brain size and neocortex as the outcome. Female weight was also included as independent variable in all analysis because it is standard procedure to control for body and thereby consider the analyses as predicting relative brain size (but see for example [13]). We did not use residuals from regressions in the analyses since it has been shown that when the independent variables are correlated this methods leads to biased estimates [79]. The mosaic brain hypothesis (see introduction) is explicitly tested when using neocortex as the outcome variable. This sums to 63 models per dependent variable (total brain and neocortex).

$$\begin{array}{cc} 2^{6}-1 & =63 \end{array}$$

It therefore follows that each predictor is included in 32 * 2 models (except female weight which was included in all).

Model selection was carried out utilizing the Akaike information criteria (AIC).

One way to assess the severity of collinearity in a least squares regression is to calculate the variance inflation factor (VIF). However, because PGLS assumes a correlated residual structure the VIF diagnostic does not carry over easily [76]. Our approach was instead to calculate posterior distributions in a Bayesian framework and visually inspect whether they correlate as an indication of collinearity (See Discussion and supporting information S1 Fig). However, for interested readers we report conventionally calculated VIF scores along with a correlation matrix and partial *R*^2^ for all variables (Supporting information S2–S5 Tables).

Research Ethics: No ethical assessment were needed because the study used published data. Animal Ethics: This study was performed without any animal subjects and no approval from ethics committees was needed.

## Results

We analyzed the effect of six predictor variables on two outcome variables: total brain size and neocortex size. First, we calculated the ‘best’ model according to the Akaike information criteria (AIC), both when using total brain size as the dependent variable and when using neocortex size as the dependent variable. As can be seen in Table 1, AIC resulted in a model that includes female weight, male group size, female group size, lifespan, female sexual maturity and fruit, omitting only innovation, as the best model predicting total brain size. Likewise, in Table 2 for neocortex size as the dependent variable, AIC resulted in a model that includes female weight, male group size, female group size and female sexual maturity.

**Table 1:** The following model was selected with AIC for total brain size as the dependent variable.

Predictor	*b*	*se*	*t*	*p*
Female weight	0.590	0.039	15.177	<0.000
Male group size	-0.090	0.058	-1.551	0.131
Female group size	0.108	0.049	2.220	0.033
Life span	0.240	0.086	2.800	0.009
Female sexual maturity	0.158	0.090	1.756	0.088
Fruit	0.238	0.088	2.720	0.010
Model summary				
R^2^	0.975			
λ	1			
*N*	40			

**Table 2:** The following model was selected with AIC for neocortex size as the dependent variable.

Predictor	*b*	*se*	*t*	*p*
Female weight	0.564	0.049	11.437	<0.000
Male group size	-0.146	0.073	-2.002	0.053
Female group size	0.233	0.059	3.958	<0.000
Life span	0.266	0.108	2.465	0.019
Female sexual maturity	0.267	0.113	2.358	0.024
Model summary				
*R*^2^	0.977			
λ	1			
*N*	40			

To get an overview of all models, i.e. the 32 models that each predictor were included in, Tables 3 and 4 illustrate the number of models in which each predictor was non-significant. Table 3 shows models that used total brain as the dependent variable, whereas Table 4 shows the same models using neocortex size as the dependent variable. As can be seen, whether a variable is a significant predictor of total brain or neocortex size depends to a worryingly high degree upon what concomitant variables that were also included in the model. Tables 5 and 6 shows the minimum and maximum p-values for each predictor depending on concomitant predictors. Most predictors range from below or close to *p* = 0.05 to high values well beyond *p* > 0.2. The only predictors that exhibit a narrow range are fruit when using total brain as the outcome, that never ranges above 0.061, and female group size when using neocortex as the outcome that never ranges above 0.015.

**Table 3:** Number of models in which each predictor was estimated non-significant (*p* > 0.05) with total brain size as the dependent variable. *N* = 40.

Predictor	Number of models in which predictor is non-significant
Female weight	0/32
Male group size	30/32
Female group size	28/32
Life span	6/32
Female sexual maturity	30/32
Innovation	32/32
Fruit	6/32

**Table 4:** Number of models in which each predictor were estimated non-significant (*p* > 0.05) with Neocortex size as the dependent variable. *N* = 40.

Predictor	Number of models in which predictor is non-significant
Female weight	0/32
Male group size	32/32
Female group size	0/32
Life span	26/32
Female sexual maturity	21/32
Innovation	32/32
Fruit	32/32

**Table 5:** Changes in p-value for each predictor when altering concomitant predictors using total brain as dependent variable. Read as follows: the focal predictor in the first column was estimated to a lowest p-value (out of all the 32 models the focal predictor where included in) shown in the second column when using concomitant predictors shown in column three. Likewise, the maximum p-value shown in column four, were estimated using concomitant predictors in column five. *N* = 40.

Focal predictor	Min p-value	Concomitant predictors	Max p-value	Concomitant predictors
Male group size	0.082	Female group size, Lifespan, Female sexual maturity, Innovation, Fruit	0.968	Lifespan
Female group size	0.017	Male group size, Lifespan, Female sexual maturity, Innovation, Fruit	0.540	Female sexual maturity, Innovation
Life span	0.004	Male group size, Female group size, Life span, Innovation	0.263	Female group size, Innovation, Fruit
Female sexual maturity	0.043	Male group size, Female group size, Life span, Innovation	0.263	Female group size, Innovation, Fruit
Innovation	0.240	Fruit	0.865	Male group size, Life span
Fruit	0.007	Male group size, Female group size, Lifespan	0.061	Male group size, Female sexual maturity

**Table 6:** The change in p-value for each predictor when altering concomitant predictors using neocortex size as dependent variable. Read as follows: the focal predictor in the first column was estimated to a lowest p-value (out of all the 32 models the focal predictor where included in) shown in the second column when using the concomitant predictors shown in column three. Likewise, the maximum p-value shown in column four, were estimated using concomitant predictors in column five. *N* = 40.

Focal predictor	Min p-value	Concomitant predictors	Max p-value	Concomitant predictors
Male group size	0.039	Female group size, Lifespan, Female sexual maturity, Innovation	0.780	Lifespan, Female sexual maturity, Innovation, Fruit
Female group size	0.0003	Male group size, Lifespan, Female sexual maturity	0.015	Female group size, Female sexual maturity, Innovation, Fruit
Life span	0.017	Male group size, Female group size, Female sexual maturity, Innovation, Fruit	0.403	Male group size, Innovation
Female sexual maturity	0.016	Male group size, Female group size, Life span, Innovation, Fruit	0.153	Male group size, Innovation
Innovation	0.074	No concomitant predictors	0.970	Male group size, Female group size, Life span
Fruit	0.138	Lifespan, Female sexual maturity, Innovation	0.699	Male group size, Female group size, Lifespan, Innovation

To further illustrate how predictors jump above and below the significance level *p* = 0.05, Table 7 shows results that extend beyond the analyses hitherto reported. Here we have reanalyzed previously reported results by systematically altering one factor at a time and observe changes in calculated p-values. The first row in Table 7 shows that the significant relationship reported between fruit in diet as the predictor and brain size as the dependent variable [35], becomes non-significant when utilizing the new, extended dataset. Row two shows the opposite change, where the reported non-significant relationship between group size and brain size [35] became significant when using other brain data (i.e. changing data from [4] to [3] & [5]). Row three shows that the reported relationship between juvenile period and brain size [40] reverses from significant to non-significant when adding more data to predictors by pooling. Row four shows that the relationship reported by Lindenfors et al. [27] between various brain parts and sexual dimorphism, female group size and male group size reverse or disappear when, again, adding more data by pooling. Lastly on row five, the significant relationship between group size and neocortex [19] had its slope significantly changed when adding more data by pooling and controlling for phylogeny. Further, ‘Dunbar’s number’, claimed to describe the cognitive threshold for group size in humans, changed from 150 to 22. But note that the 95% prediction interval for this number ranges from 0.000001 to 309, 856, 548, rendering the threshold number 150 meaningless (the asymmetry of the confidence interval stems from exponentiating the fitted *logY*). Note that when predicting with PGLS here, the model does not account for the phylogenetic position of the observation to be predicted. All reanalyzes reported here use phylogeny to correct for non-independence.

**Table 7:** Overview of changes in the relation between brain size and predictors as different data is used. *N* is identical to the original studies in all re-analyses.

Source	Reported relationship		Reanalysis changes	Result
DeCasien et al. 2017 [35]	Brain size ∼ Fruit in diet	Significant	Updated predictors^1^	Non-significant
DeCasien et al. 2017 [35]	Brain size ∼ Group size	Non-significant	Other brain data^2^	Significant
Joffe 1997 [40]	Juvenile period ∼ non visual neocortex	Significant	Updated variables^3^	Non-significant
Lindenfors et al. 2007 [27]	Specific brain parts ∼ Dimorphism, Female/male group size	Non-significant/Significant	Updated variables^4^	Significant/Non-significant
Dunbar 1992 [19]	‘Dunbar’s number’ = 150		Updated variables and control for phylogeny^5^	‘Dunbar’s number’ = 22

^1^Using [35] brain data but pooling predictors from several sources (see supporting information S6 & S7 Tables).

^2^Using predictors from [35] but changing their brain data [4] to this study’s data (i.e. pooling [3, 5], see supporting information S6 & S7 Tables).

^3^All variables used were pooled with data from [35, 40, 67](Supporting information S8 and S9 Tables).

^4^All variables used were pooled with data from [35, 67](Supporting information S10 and S11 Tables).

^5^For brain size data, we added the new data from [5] to that of [3]. Further, data on group size and body weight was pooled from [35, 67]. Dunbar’s original model was used to predict *Homo sapiens* group size (‘Dunbar’s number’), without control for phylogeny. Note that in addition to being very different from the original estimate, the new ‘Dunbar numbers’ from our more complete and phylogenetically controlled model has a huge 95% prediction interval, ranging from 0.000001 to 309, 856, 548 (Supporting information S12 and S13 Tables)

As is custom in phylogenetic comparative analysis, phylogenetic information is used to estimate the covariance of the residuals [80]. This process can lead to an *R*^2^ value different from model fit with non-phylogenetic least squares. With this in mind it can still give a crude picture of the amount of brain size variation that is explained by body size: *R*^2^ = *cor*(*predicted*, *log*[*totalbrainsize*])^2^ = 0.94, where *predicted*(*totalbrain*) = *intercept*(5.167) + *b*(0.667) * *log*(*femaleweight*).

Collinearity is member of a family of problems with model fitting referred to as weakly-identifiable parameters (or sometimes non-identifiable) [81]. If the predictors co-vary a lot, i.e. share information, their posterior parameter distributions will correlate (when *β*1 increase *β*2 must decrease and vice versa) making it hard to identify a true estimate. To investigate if our analyses suffer from collinearity we calculated both a correlation plot for all variables and the VIF scores from both OLS and PGLS models. Looking at the correlation plot it can be shown that some variables correlate substantially. However, the calculated VIF scores is not worryingly high (<10). We still believe that multicollinearity cannot be ruled out because when we calculated posterior distributions for all parameters in a Bayesian framework, for the full model (containing all six predictors) and plotted the correlation matrix (excluding varying intercepts, see supporting information S1 Fig) it is obvious that some parameters correlate substantially which would explain the varying results exposed in this study [81–83]. However, the Markov chains sampled poorly and the analyses should not be fully trusted.

## Discussion

Our analyses indicate that the field of primate brain evolution is best characterized as an array of contradicting results [1, 2] and our results reveal one reason why this is so. Within the PGLS framework, choice of what variables to include, and what observations for those variables to include, fundamentally changes the conclusions as to what drives primate brain evolution.

In this study we conducted analyses on new data [5] combined with Stephan et al.’s classic dataset on primate brain size [3] but the addition of more data did not alter the volatility in the results. If so inclined, we could have presented support for any hypothesis of our choice, but also refuted pretty much any study we would have liked. Combined with the ‘publish-or-perish’-situation in academia, this is hardly an ideal situation.

In Tables 1 and 2 we present the models that were selected with AIC. The AIC tests in turn had six explanatory variables to combine. The predictors and the AIC test itself were chosen according to our best effort to follow the established method within the field of primate brain evolution. In other words, we chose variables that according to the literature are plausible determinants in brain evolution, and used established methods to choose among combinations of predictors. If this had been a standard study, we would have moved on to discuss the biological rationale for our favorite AIC models and special attention would have been given to significant predictors (at *p* < 0.05). However, we argue that because of the breadths of hypotheses, or hierarchies of hypotheses, compatible with the results, the more important aspect of this study is the instability of the presented results (Tables 3–7).

Tables 3–6 show that most of the explanatory variables have been assigned parameter values with probability on both sides of the significance level at *p* = .05. As has happened throughout research on primate brain evolution, some researchers have concluded that one factor has been the main determinant, whereas other researchers have made different models and concluded something contradictory. Tables 3 and 4 show the most extreme cases in the exhaustive list of models. Thus, using p-values to evaluate the importance of hypotheses that affect primate brain size leaves us ambivalent. AIC was developed to select among models and thus to save us from such ambivalence, but AIC can only evaluate the models given to it, which is why the results still are dependent on pre-test variable choice.

Even though AIC has been established as praxis—a method for selecting among models and not concerned with p-values—many papers on primate brain evolution gives special status to predictors associated with *p* < .05. Following this habit, we think there is no way avoiding the problem illustrated in Tables 3–6, i.e. depending on what initial predictors happened to be included in the analyses, these predictors can either be judged important (*p* < 0.05) or non-important (*p* > 0.05). The combinatory mosaic brain hypothesis did not escape this problem of inconsistent results, as can be shown in Tables 4 and 6.

An interesting exception is female group size predicting neocortex which is always *p* < 0.05. However, it is our opinion that this result also should be judged with due caution, for several reasons. (i) In the field of primate brain evolution there are many variables that have been used to test for significant correlations. Against that background and the inherent expectation of the frequentist method to obtain error type 1 equal to *α*, it is not clear that female group size would come out significant after correcting for alpha inflation due to multiple comparisons. (ii) Many predictors in our analysis come close to the significance level at 0.05 and we see no justification to interpret those predictors as irrelevant, especially in the context of AIC where non-significant predictors is part of the best predicting model. (iii) If merely predicting neocortex size is what matters, then the best model were chosen with AIC and it makes no sense to pick one predictor, like female group size, out of that model and give it special attention.

When we included new brain data and updated variables on previously reported results, we found the same patterns. As shown in Table 7, previous results [35] indicated that brain size was best predicted by diet and not by sociality (measured as group size). When we added more observations to the explanatory variables, both diet and sociality turned out to be non-significant. When we kept their original predictors, but used pooled brain data [3, 5], sociality became significant but not diet (see supporting information S6 Table).

Further, Lindenfors et al. [27] predicted that the relative size of brain structures involved in motor skills and coordination, such as the mesencephalon, diencephalon, cerebellum and medulla oblongata, would increase in species with a larger male group size. They found male group size to be a significant predictor for all these structures except cerebellum. However, when we did a reanalysis with updated variables we found no significant relationships for any of these predictors.

The results presented here confirm a previous report where the researchers re-analyzed endocranial data [4] and concluded that “[o]ur results indicate that, even holding constant statistical methods, phylogeny, set of predictor variables, response variable data, and species sample, the behavioral and ecological correlates of brain size are sensitive to the use of different predictor datasets” ([1], p. 4).

AIC is a method for choosing the model with the lowest out-of-sample deviance and as such a method concerned with prediction, not p-values. Clearly, as shown in this paper, the best predicting model may include several variables that have non-significant p-values. In the context of AIC, it is easy to illustrate that the most predictive models sometimes do not reveal the true relationship between individual predictors and the outcome, as for example in the case of concomitant variable bias [84] or collinearity. Yet inference about individual predictors is mostly what concerns scientists of primate brain evolution, not mere prediction.

Our suggestion for future studies is to run Bayesian analysis for all regression parameters. As we have demonstrated in this study, the probability for many of the slopes given a null hypotheses lie in the region of 0.05, and as a method for decision-making—i.e. believing in a slope or not—seems unrealistically rigid and binary. Even if a Bayesian result will not give us the decisive answer we seek it will definitely provide the distributions of likelihood for each slope and, as we have initiated here, expose multicollinearity by calculating posterior distribution correlations.

Further caveats on the current practices in comparative studies of primate brain evolution have been raised by other researchers, such as problems with measuring and comparing intelligence [15, 85], the idea of adaptive specializations of cognitive mechanisms [18, 86], validity of observational data versus experiment [18], choice of brain measure [6, 52], measuring and defining sociality [70, 87, 88] and p-hacking: given that the same sample on brain volume [3] has been modeled against many variables, it is to be expected that Type 1-errors will emerge [89]. Also, there is some evidence that different data samples are qualitatively different from each other [70, 90, 91]. It has for example been shown that data on body size often are averaged, inaccurate and from unspecified sources [90, 92].

In our analyses, variation in body size explain 94% of the variation in brain size. This does not leave much to be explained by the competing adaptive hypotheses. The fact that these adaptive hypotheses explain very little variation, taken together with the unstable nature of results, suggest that it is easy to overstate the importance of sociality, diet, problem solving, or life-history for understanding brain evolution.

Further, other measures not included here may be more important for our understanding of brain evolution. Indeed, other combinations of predictors have been used in previous studies, however, we believe that adding more predictors would reveal similar inconsistencies in the results and that the six predictors used in this study suffice to illustrate this. That variation in sensory and perceptual systems give rise to variation in brain size is not controversial [93, 94]. A primate with very large eyes will have brain areas that correspond to sensory and perceptual needs. In addition, animals that are motor flexible, have many different kinds of muscles, and large behavior repertoires need brain areas that control muscles. Therefore, larger brains are needed to drive more motor flexible bodies [95]. To put this in Tinbergian terminology: a mechanistic link between brain size and body functions is straight forward and non-controversial, while a functional link between brain size and mental capacities is harder to define to non-controversial precision.

We conclude that, given the instability of results and the PGLS approach, there is no empirical justification to highlight any particular hypothesis of those adaptive hypotheses we have examined here, as the main determinant of primate brain evolution.

## Supporting information

S1 Fig Correlation matrix of posterior distributions for all predictors used in this study calculated using a bayesian multilevel model. (DOCX)

S1 Table Main data set. (DOCX)

S2 Table VIF scores using Ordinary Least Squares (OLS) and Phylogenetic Generalized Least Squares (PGLS). (DOCX)

S3 Table Correlation matrix all variable. (DOCX)

S4 Table Partial R2. (DOCX)

S5 Table Partial R2. (DOCX)

S6 Table Reevaluating DeCasien [35] et al.’s reported relationship between brain weight, percent frugivory and group size, with three alternative statistical models. (DOCX)

S7 Table Data used to reevaluate DeCasien et al. [35]. (DOCX)

S8 Table Reevaluating Joffe [40] results of a significant relationships between juvenile period and the ratio of non-visual cortex to the rest of the brain. (DOCX)

S9 Table Data used to reevaluate Joffe [40]. (DOCX)

S10 Table Reevaluating Lindenfors [27]. (DOCX)

S11 Table Data used to reevaluate Lindenfors [27]. (DOCX)

S12 Table Dunbar’s number [19]. (DOCX)

S13 Table Data used to reevaluate Dunbar [19]. (DOCX)
