# Supplementary material for: Whatever you want: Inconsistent results are the rule, not the exception, in the study of primate brain evolution
Source: PLoS One. 2019 Jul 22;14(7):e0218655. doi: 10.1371/journal.pone.0218655 (PMC6645455; doi:10.1371/journal.pone.0218655)
Supplement: S1 Fig — (DOCX) [file pone.0218655.s001.docx]

| Figure S1. Correlation matrix of posterior distributions for all predictors used in this study calculated using a bayesian multilevel model. Total brain was used as the dependent variable. Several predictors correlate, for example male group size/female group size and lifespan/innovation. This can be interpreted to mean that the PGLS normally used will report spurious point estimates from these distributions. |
| --- |
| 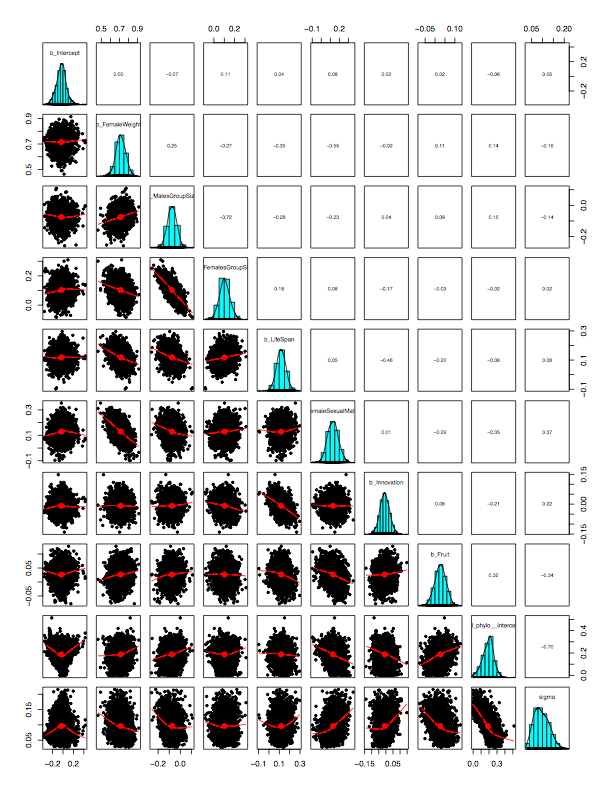 |
