# Supplementary material for: Whatever you want: Inconsistent results are the rule, not the exception, in the study of primate brain evolution
Source: PLoS One. 2019 Jul 22;14(7):e0218655. doi: 10.1371/journal.pone.0218655 (PMC6645455; doi:10.1371/journal.pone.0218655)
Supplement: S1 Table — (DOCX) [file pone.0218655.s002.docx]

| Table S1. Main data set | | | | | | | | | |
| --- | --- | --- | --- | --- | --- | --- | --- | --- | --- |
| *Species* | *Total brain* | *Neocortex* | *Female weight* | *Male group size* | *Female group size* | *Life span* | *Female sexual maturity* | *Fruit* | *Innovation* |
| *Aotus trivirgatus* | 16557.540 | 9812.740 | 834.500 | 1.100 | 1.000 | 20.000 | 30.000 | 54.833 | 0.000 |
| *Ateles geoffroyi* | 101034.000 | 70856.000 | 7449.500 | 4.000 | 17.000 | 27.300 | 72.000 | 75.233 | 0.000 |
| *Callicebus moloch* | 17944.000 | 11163.000 | 956.000 | 1.000 | 1.000 | 12.000 | 30.000 | 54.340 | 0.000 |
| *Callimico goeldii* | 11311.170 | 6333.720 | 476.500 | 1.000 | 2.000 | 17.900 | 8.500 | 29.000 | 1.000 |
| *Callithrix jacchus* | 7682.880 | 4456.490 | 324.000 | 2.700 | 2.900 | 11.700 | 22.000 | 15.475 | 1.000 |
| *Callithrix pygmaea* | 4305.170 | 2388.060 | 100.500 | 1.500 | 1.000 | 11.670 | 24.000 | 30.000 | 1.000 |
| *Cebus apella* | 74229.030 | 37879.400 | 2504.500 | 3.200 | 4.900 | 44.000 | 56.000 | 48.400 | 39.000 |
| *Cercopithecus ascanius* | 63505.000 | 45166.000 | 2910.500 | 1.000 | 8.500 | 22.500 | 54.000 | 55.900 | 1.000 |
| *Cercopithecus mitis* | 68503.930 | 42414.190 | 4495.500 | 1.100 | 8.800 | 20.000 | 70.500 | 45.813 | 4.000 |
| *Cercopithecus nictitans* | 73183.470 | 37132.310 | 4260.000 | 1.000 | 2.000 | 31.000 | 48.000 | 69.550 | 0.000 |
| *Colobus guereza* | 77247.810 | 33524.270 | 8353.000 | 1.300 | 2.900 | 22.250 | 48.700 | 18.711 | 1.000 |
| *Daubentonia madagascariensis* | 42611.000 | 22127.000 | 2490.000 | 1.000 | 1.000 | 22.300 | 29.000 | 0.000 | 4.000 |
| *Erythrocebus patas* | 100086.400 | 63082.540 | 5700.000 | 1.200 | 10.400 | 21.580 | 41.200 | 39.374 | 1.000 |
| *Eulemur fulvus* | 22106.000 | 12207.000 | 2205.000 | 3.200 | 3.500 | 30.080 | 22.000 | 36.227 | 3.000 |
| *Galago demidoff* | 3203.000 | 1568.000 | 64.500 | 1.000 | 1.000 | 13.000 | 8.000 | 18.333 | 0.000 |
| *Galago senegalensis* | 4043.450 | 1906.950 | 193.500 | 1.000 | 1.000 | 16.500 | 6.700 | 0.000 | 0.000 |
| *Gorilla gorilla gorilla* | 435339.268 | 255646.545 | 77422.000 | 1.800 | 4.400 | 50.000 | 78.000 | 26.371 | 25.000 |
| *Hylobates lar* | 97505.000 | 65800.000 | 5360.500 | 1.000 | 1.000 | 31.500 | 108.000 | 61.425 | 0.000 |
| *Lagothrix lagotricha* | 93589.850 | 56371.030 | 7020.000 | 5.800 | 8.200 | 25.920 | 96.000 | 71.540 | 0.000 |
| *Lophocebus albigena* | 93706.710 | 56812.610 | 5998.000 | 4.800 | 7.800 | 32.700 | 48.000 | 49.643 | 0.000 |
| *Loris tardigradus* | 6269.000 | 3524.000 | 193.000 | 1.000 | 1.000 | 12.000 | 13.000 | 15.000 | 1.000 |
| *Macaca fascicularis* | 53845.390 | 26848.310 | 3554.000 | 4.600 | 10.600 | 37.080 | 51.600 | 63.450 | 7.000 |
| *Macaca fuscata* | 89294.870 | 48310.080 | 8030.000 | 7.300 | 17.500 | 33.000 | 54.000 | 35.333 | 26.000 |
| *Macaca mulatta* | 88121.580 | 54019.950 | 6377.500 | 9.100 | 23.800 | 29.000 | 42.000 | 55.000 | 5.000 |
| *Macaca nemestrina* | 94813.840 | 42872.640 | 6119.500 | 2.400 | 14.900 | 26.290 | 35.000 | 72.500 | 1.000 |
| *Macaca sylvanus* | 83284.630 | 45352.780 | 10000.000 | 5.200 | 7.000 | 22.000 | 46.000 | 28.500 | 0.000 |
| *Microcebus murinus* | 1688.150 | 751.510 | 63.000 | 1.000 | 1.000 | 15.420 | 9.500 | 51.000 | 0.000 |
| *Miopithecus talapoin* | 37776.000 | 26427.000 | 1560.000 | 13.000 | 21.300 | 27.670 | 48.000 | 57.460 | 0.000 |
| *Nasalis larvatus* | 92797.000 | 62685.000 | 9775.000 | 1.000 | 3.700 | 13.500 | 48.000 | 32.766 | 0.000 |
| *Nycticebus coucang* | 11755.000 | 6192.000 | 588.500 | 1.000 | 1.000 | 16.000 | 19.000 | 53.333 | 0.000 |
| *Otolemur crassicaudatus* | 9668.000 | 4723.000 | 1018.000 | 1.000 | 1.000 | 15.000 | 12.000 | 24.075 | 2.000 |
| *Pan paniscus* | 306268.190 | 143537.040 | 33200.000 | 7.000 | 8.900 | 40.000 | 102.000 | 52.000 | 10.000 |
| *Pan troglodytes troglodytes* | 357703.890 | 197764.970 | 40367.000 | 6.700 | 12.400 | 53.000 | 126.000 | 67.240 | 321.000 |
| *Papio anubis* | 190957.000 | 140142.000 | 13734.500 | 9.100 | 17.500 | 45.000 | 57.500 | 26.657 | 12.000 |
| *Papio hamadryas* | 168266.300 | 85309.200 | 10325.000 | 6.500 | 8.200 | 35.600 | 51.500 | 88.000 | 6.000 |
| *Perodicticus potto* | 13212.000 | 6683.000 | 852.000 | 1.000 | 1.000 | 22.330 | 8.000 | 67.725 | 0.000 |
| *Pongo pygmaeus* | 323450.540 | 164492.870 | 36324.000 | 1.000 | 1.000 | 57.330 | 84.000 | 65.333 | 53.000 |
| *Propithecus verreauxi* | 25194.000 | 13170.000 | 2950.000 | 3.000 | 2.800 | 18.170 | 30.000 | 29.333 | 0.000 |
| *Saimiri sciureus* | 22107.280 | 13865.510 | 722.500 | 2.700 | 7.900 | 21.000 | 30.000 | 38.233 | 3.000 |
| *Varecia variegata variegata* | 29713.000 | 15293.000 | 3255.000 | 2.500 | 3.000 | 13.000 | 20.000 | 73.950 | 0.000 |
