# Supplementary material for: Whatever you want: Inconsistent results are the rule, not the exception, in the study of primate brain evolution
Source: PLoS One. 2019 Jul 22;14(7):e0218655. doi: 10.1371/journal.pone.0218655 (PMC6645455; doi:10.1371/journal.pone.0218655)
Supplement: S2 Table — (DOCX) [file pone.0218655.s003.docx]

| Table S2. VIF scores using Ordinary Least Squares (OLS) and Phylogenetic Generalized Least Squares (PGLS) | | |
| --- | --- | --- |
|  | *OLS* | *PGLS* |
| *Female weight* | *4.424* | *4.191* |
| *Male group size* | *3.082* | *2.665* |
| *Female group size* | *3.490* | *2.420* |
| *Lifespan* | *3.041* | *2.767* |
| *Female sexual maturity* | *3.825* | *3.178* |
| *Innovation* | *2.189* | *1.574* |
| *Fruit* | *1.382* | *1.050* |
