# Supplementary material for: Whatever you want: Inconsistent results are the rule, not the exception, in the study of primate brain evolution
Source: PLoS One. 2019 Jul 22;14(7):e0218655. doi: 10.1371/journal.pone.0218655 (PMC6645455; doi:10.1371/journal.pone.0218655)
Supplement: S3 Table — (DOCX) [file pone.0218655.s004.docx]

| Table S3. Correlation matrix all variable | | | | | | | | | |
| --- | --- | --- | --- | --- | --- | --- | --- | --- | --- |
|  | *Total brain* | *Neocortex* | *Female weight* | *Male group size* | *Female group size* | *Lifespan* | *Female sexual maturity* | *Innovation* | *Fruit* |
| *Total brain* | *1.000* | *0.996* | *0.977* | *0.469* | *0.624* | *0.710* | *0.885* | *0.554* | *0.396* |
| *Neocortex* | *0.996* | *1.000* | *0.967* | *0.475* | *0.642* | *0.686* | *0.895* | *0.530* | *0.397* |
| *Female weight* | *0.977* | *0.967* | *1.000* | *0.447* | *0.575* | *0.675* | *0.833* | *0.529* | *0.356* |
| *Male group size* | *0.469* | *0.475* | *0.447* | *1.000* | *0.800* | *0.486* | *0.479* | *0.350* | *0.299* |
| *Female group size* | *0.624* | *0.642* | *0.575* | *0.800* | *1.000* | *0.448* | *0.590* | *0.330* | *0.351* |
| *Lifespan* | *0.710* | *0.686* | *0.675* | *0.486* | *0.448* | *1.000* | *0.553* | *0.700* | *0.310* |
| *Female sexual maturity* | *0.885* | *0.895* | *0.833* | *0.479* | *0.590* | *0.553* | *1.000* | *0.472* | *0.434* |
| *Innovation* | *0.554* | *0.530* | *0.529* | *0.350* | *0.330* | *0.700* | *0.472* | *1.000* | *0.065* |
| *Fruit* | *0.396* | *0.397* | *0.356* | *0.299* | *0.351* | *0.310* | *0.434* | *0.065* | *1.000* |
