# Supplementary material for: Whatever you want: Inconsistent results are the rule, not the exception, in the study of primate brain evolution
Source: PLoS One. 2019 Jul 22;14(7):e0218655. doi: 10.1371/journal.pone.0218655 (PMC6645455; doi:10.1371/journal.pone.0218655)
Supplement: S4 Table — (DOCX) [file pone.0218655.s005.docx]

| Table S4. Partial R2. This table shows partial R2 by comparing the full model, including all seven predictors, to a reduced model excluding one predictor of interest adjusting for correlated errors. The right column shows the percentage of variation that a reduced model cannot explain but can be explained by a full model: SSRreduced-SSRfull/SSRreduced. These partial R2 were calculated using total brain as the dependent variable. | |
| --- | --- |
| Predictor | Partial *R*^2^ |
| Female weight | 0.876 |
| Female group size | 0.164 |
| Male group size | 0.091 |
| Max life span | 0.222 |
| Female sexual maturity | 0.116 |
| Fruit | 0.191 |
| Innovation | 0.040 |
