# Supplementary material for: Whatever you want: Inconsistent results are the rule, not the exception, in the study of primate brain evolution
Source: PLoS One. 2019 Jul 22;14(7):e0218655. doi: 10.1371/journal.pone.0218655 (PMC6645455; doi:10.1371/journal.pone.0218655)
Supplement: S6 Table — (DOCX) [file pone.0218655.s007.docx]

| Table S6. Reevaluating DeCasien [35] et al.’s reported relationship between brain weight, percent frugivory and group size, with three alternative statistical models. In the first model, we used [35] original data for brain weight and predictors pooled from [35, 51, 67, 68]. In contrast to [35], we found that percent frugivory was not a significant predictor for brain weight. In the second model, we used [35] original predictors and the pooled brain weight from [3] and [5], and fixing lambda (λ) = 1. Now we found that group size was significant but not percent frugivory. In the third model, lambda (λ) was estimated, thereby impacting the estimated parameters grossly, making percent frugivory significant, but not group size. | | | | |
| --- | --- | --- | --- | --- |
| *Brain weight^1^ ~ Body weight + Group size + Fruit^2^* | | | | |
| *Predictor* | *b* | *se* | *t* | *p* |
| *Body weight* | 0.629 | 0.026 | 24.291 | <0.000 |
| *Group size* | 0.019 | 0.024 | 0.796 | 0.428 |
| *Fruit* | 0.051 | 0.066 | 0.765 | 0.446 |
| *Model summary* |  |  |  |  |
| *R^2^* | 0.973 |  |  |  |
| *λ* | 0.948 |  |  |  |
| *Brain weight^3^ ~ Body weight + Group size + Fruit^4^* | | | | |
| *Predictor* | *b* | *se* | *t* | *p* |
| *Body weight* | 0.593 | 0.039 | 15.213 | <0.000 |
| *Group size* | 0.117 | 0.057 | 2.043 | 0.048 |
| *Fruit* | 0.113 | 0.089 | 1.274 | 0.211 |
| *Model summary* |  |  |  |  |
| *R^2^* | 0.941 |  |  |  |
| *λ* | 1.000 |  |  |  |
| *Brain weight^3^ ~ Body weight + Group size + Fruit^4^* | | | | |
| *Predictor* | *b* | *se* | *t* | *p* |
| *Body weight* | 0.589 | 0.039 | 15.218 | <0.000 |
| *Group size* | 0.115 | 0.057 | 2.009 | 0.052 |
| *Fruit* | 0.121 | 0.017 | 6.912 | <0.000 |
| *Model summary* |  |  |  |  |
| *R^2^* | 0.941 |  |  |  |
| *λ* | 1.018 |  |  |  |
| ^1^Brain weight taken from (35). ^2^Predictors pooled from (35, 51, 67, 68). ^3^Brain weight pooled from (3, 5). ^4^Predictors from (35). | | | | |
