# Supplementary material for: Whatever you want: Inconsistent results are the rule, not the exception, in the study of primate brain evolution
Source: PLoS One. 2019 Jul 22;14(7):e0218655. doi: 10.1371/journal.pone.0218655 (PMC6645455; doi:10.1371/journal.pone.0218655)
Supplement: S7 Table — (DOCX) [file pone.0218655.s008.docx]

| Table S7. Data used to reevaluate DeCasien et al. [35] | | | | |
| --- | --- | --- | --- | --- |
| *Species* | *Brain weight* | *Weight* | *Group size* | *Fruit* |
| *Alouatta caraya* | 52.720 | 5474.577 | 7.792 | 23.200 |
| *Alouatta palliata* | 50.908 | 6459.873 | 14.474 | 37.020 |
| *Alouatta seniculus* | 56.570 | 6139.555 | 6.933 | 50.550 |
| *Aotus trivirgatus* | 17.213 | 847.275 | 3.706 | 54.833 |
| *Arctocebus calabarensis* | 7.171 | 279.168 | 1.125 | 13.333 |
| *Ateles belzebuth* | 118.576 | 7318.820 | 19.950 | 82.800 |
| *Ateles geoffroyi* | 108.535 | 7163.275 | 35.009 | 75.233 |
| *Ateles paniscus* | 105.941 | 8282.264 | 26.900 | 84.767 |
| *Avahi laniger* | 10.251 | 1237.500 | 2.833 | 0.000 |
| *Brachyteles arachnoides* | 120.100 | 9043.750 | 24.792 | 42.346 |
| *Bunopithecus hoolock* | 114.665 | 6764.750 | 3.375 | 68.250 |
| *Cacajao calvus* | 73.300 | 3229.010 | 22.500 | 19.100 |
| *Callicebus moloch* | 17.725 | 935.903 | 3.633 | 54.340 |
| *Callicebus torquatus* | 20.559 | 1198.750 | 3.538 | 55.100 |
| *Callimico goeldii* | 11.752 | 513.125 | 6.250 | 29.000 |
| *Callithrix argentata* | 8.046 | 353.062 | 7.850 | 33.000 |
| *Callithrix geoffroyi* | 8.643 | 350.500 | 9.000 | 24.000 |
| *Callithrix jacchus* | 7.644 | 296.603 | 8.139 | 15.475 |
| *Callithrix penicillata* | 7.555 | 325.120 | 6.950 | 30.000 |
| *Callithrix pygmaea* | 4.452 | 115.532 | 5.964 | 30.000 |
| *Cebus albifrons* | 64.715 | 2592.030 | 20.613 | 48.300 |
| *Cebus apella* | 68.747 | 2873.230 | 13.980 | 48.400 |
| *Cebus capucinus* | 73.786 | 2941.179 | 16.484 | 65.175 |
| *Cebus olivaceus* | 72.963 | 2736.190 | 20.710 | 45.333 |
| *Cercocebus agilis* | 118.320 | 7566.405 | 15.000 | 76.000 |
| *Cercocebus atys* | 99.806 | 8185.310 | 65.000 | 84.750 |
| *Cercocebus galeritus* | 102.637 | 7636.250 | 27.050 | 77.200 |
| *Cercocebus torquatus* | 111.434 | 7862.401 | 28.483 | 77.500 |
| *Cercopithecus ascanius* | 63.615 | 3730.998 | 25.147 | 55.900 |
| *Cercopithecus campbelli* | 58.982 | 3668.455 | 12.063 | 78.150 |
| *Cercopithecus cephus* | 67.925 | 3550.813 | 9.375 | 61.060 |
| *Cercopithecus diana* | 64.862 | 4534.818 | 21.550 | 49.450 |
| *Cercopithecus lhoesti* | 76.000 | 4856.868 | 15.800 | 33.250 |
| *Cercopithecus mitis* | 73.959 | 6215.840 | 22.288 | 45.813 |
| *Cercopithecus neglectus* | 67.543 | 5705.920 | 7.700 | 72.650 |
| *Cercopithecus nictitans* | 73.686 | 5321.568 | 15.625 | 69.550 |
| *Cercopithecus pogonias* | 63.268 | 3584.045 | 13.917 | 67.410 |
| *Chiropotes satanas* | 50.070 | 2920.068 | 18.400 | 40.744 |
| *Chlorocebus aethiops* | 63.924 | 4305.373 | 25.035 | 56.435 |
| *Colobus angolensis* | 71.564 | 8751.328 | 9.950 | 11.450 |
| *Colobus guereza* | 77.371 | 10489.438 | 8.400 | 18.711 |
| *Colobus polykomos* | 76.020 | 8910.396 | 11.900 | 20.500 |
| *Colobus satanas* | 80.200 | 9830.000 | 13.367 | 9.950 |
| *Daubentonia madagascariensis* | 46.345 | 2579.062 | 2.375 | 0.000 |
| *Erythrocebus patas* | 97.872 | 8454.075 | 27.360 | 39.374 |
| *Eulemur coronatus* | 21.394 | 1877.463 | 7.675 | 45.000 |
| *Eulemur fulvus* | 24.786 | 2217.415 | 9.490 | 36.227 |
| *Eulemur macaco macaco* | 24.634 | 2388.405 | 9.283 | 45.000 |
| *Eulemur mongoz* | 21.452 | 1715.188 | 3.100 | 24.375 |
| *Eulemur rubriventer* | 26.924 | 1800.900 | 3.150 | 31.000 |
| *Euoticus elegantulus* | 5.734 | 288.747 | 1.750 | 6.500 |
| *Galago alleni* | 6.100 | 261.500 | 2.375 | 75.000 |
| *Galago demidoff* | 3.380 | 62.993 | 2.625 | 18.333 |
| *Galago moholi* | 3.839 | 178.490 | 2.500 | 0.000 |
| *Galago senegalensis* | 4.130 | 210.331 | 1.000 | 0.000 |
| *Galago zanzibaricus* | 3.641 | 145.175 | 3.250 | 26.750 |
| *Gorilla gorilla gorilla* | 507.247 | 122360.748 | 12.874 | 26.371 |
| *Hapalemur griseus* | 14.600 | 983.167 | 3.775 | 3.050 |
| *Hylobates agilis* | 90.648 | 5550.680 | 4.175 | 63.667 |
| *Hylobates klossii* | 92.043 | 5828.073 | 3.500 | 71.333 |
| *Hylobates lar* | 105.276 | 5546.560 | 3.757 | 61.425 |
| *Hylobates moloch* | 106.708 | 6224.703 | 3.400 | 60.667 |
| *Hylobates muelleri* | 90.893 | 5723.703 | 3.425 | 61.000 |
| *Hylobates pileatus* | 87.734 | 5554.343 | 4.425 | 74.133 |
| *Indri indri* | 36.309 | 8417.500 | 3.890 | 27.100 |
| *Lagothrix lagotricha* | 99.688 | 7398.948 | 21.055 | 71.540 |
| *Lemur catta* | 23.246 | 2326.497 | 16.525 | 54.182 |
| *Leontopithecus chrysomelas* | 12.262 | 616.200 | 6.700 | 70.150 |
| *Leontopithecus rosalia* | 13.116 | 606.255 | 6.300 | 71.075 |
| *Lepilemur mustelinus* | 9.904 | 701.000 | 1.250 | 5.500 |
| *Lophocebus albigena* | 97.489 | 7528.118 | 18.640 | 49.643 |
| *Loris tardigradus* | 6.052 | 231.153 | 1.500 | 15.000 |
| *Macaca fascicularis* | 66.530 | 4550.553 | 29.411 | 63.450 |
| *Macaca fuscata* | 98.593 | 9506.940 | 60.075 | 35.333 |
| *Macaca mulatta* | 88.941 | 7168.990 | 52.431 | 55.000 |
| *Macaca nemestrina* | 109.871 | 7514.672 | 38.430 | 72.500 |
| *Macaca nigra* | 91.456 | 7671.098 | 41.000 | 67.000 |
| *Macaca radiata* | 79.466 | 5569.498 | 31.280 | 51.500 |
| *Macaca sinica* | 73.142 | 4476.498 | 23.000 | 72.333 |
| *Macaca sylvanus* | 97.365 | 12086.633 | 25.530 | 28.500 |
| *Mandrillus leucophaeus* | 185.962 | 16251.650 | 55.250 | 71.000 |
| *Mandrillus sphinx* | 162.409 | 21046.265 | 54.450 | 81.738 |
| *Microcebus murinus* | 1.721 | 62.467 | 3.050 | 51.000 |
| *Miopithecus talapoin* | 37.700 | 1493.477 | 88.838 | 57.460 |
| *Nasalis larvatus* | 96.211 | 14264.663 | 9.470 | 32.766 |
| *Nomascus concolor* | 131.700 | 7168.368 | 4.900 | 21.000 |
| *Nycticebus coucang* | 10.729 | 736.050 | 1.000 | 53.333 |
| *Nycticebus pygmaeus* | 7.534 | 416.387 | 1.000 | 50.000 |
| *Otolemur crassicaudatus* | 12.201 | 1235.602 | 2.625 | 24.075 |
| *Otolemur garnettii* | 11.239 | 960.793 | 3.000 | 40.900 |
| *Pan paniscus* | 346.237 | 38311.238 | 46.750 | 52.000 |
| *Pan troglodytes troglodytes* | 376.125 | 46647.500 | 38.854 | 67.240 |
| *Papio anubis* | 171.798 | 17825.885 | 52.323 | 26.657 |
| *Papio cynocephalus* | 169.408 | 15773.483 | 54.003 | 32.850 |
| *Papio hamadryas* | 154.438 | 13319.930 | 36.313 | 88.000 |
| *Papio papio* | 165.300 | 17811.025 | 136.667 | 48.500 |
| *Papio ursinus* | 195.433 | 21157.360 | 41.900 | 39.600 |
| *Perodicticus potto* | 12.846 | 982.746 | 2.125 | 67.725 |
| *Piliocolobus badius* | 65.968 | 8060.375 | 30.240 | 12.838 |
| *Pithecia pithecia* | 33.352 | 1696.298 | 2.675 | 57.700 |
| *Pongo pygmaeus* | 382.457 | 56270.573 | 2.300 | 65.333 |
| *Presbytis comata* | 61.124 | 6577.500 | 7.000 | 24.500 |
| *Presbytis melalophos* | 66.905 | 5940.312 | 12.083 | 33.580 |
| *Propithecus verreauxi* | 27.049 | 3244.710 | 6.710 | 29.333 |
| *Pygathrix nemaeus* | 91.956 | 9485.183 | 15.375 | 15.000 |
| *Saguinus fuscicollis* | 8.147 | 385.415 | 5.350 | 52.133 |
| *Saguinus geoffroyi* | 14.129 | 497.950 | 6.025 | 47.500 |
| *Saguinus midas* | 10.123 | 530.102 | 5.700 | 63.075 |
| *Saguinus mystax* | 11.523 | 532.734 | 5.550 | 51.733 |
| *Saimiri boliviensis* | 25.740 | 759.113 | 40.000 | 21.500 |
| *Saimiri oerstedii* | 23.108 | 729.250 | 26.600 | 25.000 |
| *Saimiri sciureus* | 24.142 | 811.120 | 33.654 | 38.233 |
| *Semnopithecus entellus* | 113.780 | 12066.757 | 27.025 | 26.467 |
| *Symphalangus syndactylus* | 128.889 | 10278.792 | 3.930 | 43.775 |
| *Tarsius bancanus* | 3.250 | 111.415 | 2.500 | 0.000 |
| *Tarsius syrichta* | 3.480 | 116.518 | 1.500 | 0.000 |
| *Theropithecus gelada* | 136.207 | 15377.893 | 80.650 | 14.500 |
| *Trachypithecus cristatus* | 59.148 | 5856.968 | 27.725 | 18.000 |
| *Trachypithecus johnii* | 86.482 | 11349.180 | 12.700 | 23.667 |
| *Trachypithecus obscurus* | 63.663 | 6491.105 | 13.650 | 35.575 |
| *Trachypithecus phayrei* | 76.736 | 7673.180 | 10.850 | 24.400 |
| *Trachypithecus pileatus* | 107.372 | 10726.655 | 8.050 | 26.133 |
| *Trachypithecus vetulus* | 63.499 | 7270.722 | 10.250 | 34.000 |
| *Varecia variegata variegata* | 32.639 | 3348.750 | 6.217 | 73.950 |
