# Supplementary material for: Whatever you want: Inconsistent results are the rule, not the exception, in the study of primate brain evolution
Source: PLoS One. 2019 Jul 22;14(7):e0218655. doi: 10.1371/journal.pone.0218655 (PMC6645455; doi:10.1371/journal.pone.0218655)
Supplement: S8 Table — (DOCX) [file pone.0218655.s009.docx]

| Table S8. Reevaluating Joffe [40] results of a significant relationships between juvenile period1 and the ratio of non-visual cortex to the rest of the brain^2^. These relations turn non-significant when using pooled brain data [3, 5] and pooling juvenile period and weight data [35, 67]. | | | | |
| --- | --- | --- | --- | --- |
| *Juvenile period^1^ ~ Non visual neocortex ratio* | | | | |
|  | *b* | *se* | *t* | *p* |
| *Non visual neocortex ratio^2^* | 0.121 | 0.075 | 1.604 | 0.125 |
| *Model summary:* |  |  |  |  |
| *R^2^* | 0.281 |  |  |  |
| *λ* | 0.404 |  |  |  |
| *Juvenile period^1^ ~ Non visual neocortex ratio + Weight* | | | | |
|  | *b* | *se* | *t* | *p* |
| *Non visual neocortex ratio* | 0.106 | 0.084 | 1.261 | 0.222 |
| *Weight* | 0.001 | 0.001 | 0.482 | 0.635 |
| *Model summary:* |  |  |  |  |
| *R^2^* | 0.285 |  |  |  |
| *λ* | 0.397 |  |  |  |
| ^1^Juvenile period is calculated as the ratio (age till sexual maturity minus weaning period) / lifespan. ^2^Non visual neocortex ratio is calculated as (neocortex minus visual cortex) / rest of brain. | | | | |
