# Supplementary material for: Whatever you want: Inconsistent results are the rule, not the exception, in the study of primate brain evolution
Source: PLoS One. 2019 Jul 22;14(7):e0218655. doi: 10.1371/journal.pone.0218655 (PMC6645455; doi:10.1371/journal.pone.0218655)
Supplement: S9 Table — (DOCX) [file pone.0218655.s010.docx]

| Table S9. Data used to reevaluate Joffe [40] | | | |
| --- | --- | --- | --- |
| *Species* | *Juvenile period* | *Non-visual*  *neocortex ratio* | *Weight (g)* |
| *Aotus trivirgatus* | 0.114 | 1.285 | 847.275 |
| *Ateles geoffroyi* | 0.135 | 2.191 | 7163.275 |
| *Callimico goeldii* | 0.029 | 1.270 | 513.125 |
| *Callithrix jacchus* | 0.135 | 1.167 | 296.603 |
| *Callithrix pygmaea* | 0.149 | 1.250 | 115.532 |
| *Eulemur fulvus fulvus* | 0.048 | 1.079 | 2217.415 |
| *Galago senegalensis* | 0.042 | 0.814 | 62.993 |
| *Galagoides demidoff* | 0.017 | 0.736 | 210.331 |
| *Gorilla gorilla gorilla* | 0.074 | 1.338 | 122360.748 |
| *Homo sapiens* | 0.145 | 4.010 | 58500.000 |
| *Lagothrix lagotricha* | 0.275 | 1.346 | 7398.948 |
| *Lophocebus albigena* | 0.104 | 1.355 | 7528.118 |
| *Loris tardigradus* | 0.051 | 1.070 | 231.153 |
| *Macaca mulatta* | 0.090 | 1.391 | 7168.990 |
| *Microcebus murinus* | 0.044 | 0.643 | 62.467 |
| *Miopithecus talapoin* | 0.126 | 2.060 | 1493.477 |
| *Nycticebus coucang* | 0.066 | 0.965 | 736.050 |
| *Otolemur crassicaudatus* | 0.041 | 0.840 | 1235.602 |
| *Pan troglodytes troglodytes* | 0.121 | 1.145 | 46647.500 |
| *Perodicticus potto* | 0.003 | 0.939 | 982.746 |
| *Saguinus oedipus* | 0.097 | 1.145 | 405.048 |
