# Supplementary material for: Whatever you want: Inconsistent results are the rule, not the exception, in the study of primate brain evolution
Source: PLoS One. 2019 Jul 22;14(7):e0218655. doi: 10.1371/journal.pone.0218655 (PMC6645455; doi:10.1371/journal.pone.0218655)
Supplement: S10 Table — (DOCX) [file pone.0218655.s011.docx]

| Table S10. Reevaluating Lindenfors [27]. Each model below predicts the relative size of a specific brain part using body size dimorphism, male group size and female group size. In the original paper predictors, based on contrasts, were sequentially removed, keeping those with p ≤ 0.1. Here we have used PGLS and AIC to choose model with updated data^2^. The original Lindenfors et al. [27] results are shown in parenthesis for comparison. | | | | |
| --- | --- | --- | --- | --- |
| *Telencephalon ~ Rest of brain^1^ (Rest of brain , Dimorphism, Male group size, Female group size)* | | | | |
|  | *b* | *se* | *t* | *p* |
| *Rest of brain* | 1.061 | 0.059 | 17.992 | <0.000 |
| *Model summary* |  |  |  |  |
| *R^2^* | 0.924 |  |  |  |
| *λ* | 1.003 |  |  |  |
| *Neocortex ~ Rest of brain + Female group size (Rest of brain, Male group size, Female group size)* | | | | |
|  | *b* | *se* | *t* | *p* |
| *Rest of brain* | 0.723 | 0.087 | 8.318 | <0.000 |
| *Female group size* | 0.324 | 0.069 | 4.706 | <0.000 |
| *Model summary* |  |  |  |  |
| *R^2^* | 0.939 |  |  |  |
| *λ* | 1.000 |  |  |  |
| *Hippocampus ~ Rest of brain + Dimorphism + Male group size (Rest of brain, Female group size)* | | | | |
|  | *b* | *se* | *t* | *p* |
| *Rest of brain* | 0.821 | 0.066 | 12.394 | <0.000 |
| *Dimorphism* | -0.851 | 0.279 | -3.053 | 0.004 |
| *Male group size* | -0.125 | 0.070 | -1.802 | 0.079 |
| *Model summary* |  |  |  |  |
| *R^2^* | 0.842 |  |  |  |
| *λ* | 0.680 |  |  |  |
| *Diencephalon ~ Rest of brain + Dimorphism (Rest of brain , Dimorphism, Male group size, Female group size)* | | | | |
|  | *b* | *se* | *t* | *p* |
| *Rest of brain* | 0.887 | 0.019 | 45.941 | <0.000 |
| *Dimorphism* | -0.116 | 0.081 | -1.434 | 0.159 |
| *Model summary* |  |  |  |  |
| *R^2^* | 0.989 |  |  |  |
| *λ* | 0.716 |  |  |  |
| *Cerebellum ~ Rest of brain (Rest of brain)* | | | | |
|  | *b* | *se* | *t* | *p* |
| *Rest of brain* | 1.000 | 0.030 | 33.654 | <0.000 |
| *Model summary* |  |  |  |  |
| *R^2^* | 0.965 |  |  |  |
| *λ* | 0.926 |  |  |  |
| *Mesencephalon ~ Rest of brain (Rest of brain, Dimorphism)* | | | | |
|  | *b* | *se* | *t* | *p* |
| *Rest of brain* | 0.651 | 0.027 | 24.479 | <0.000 |
| *Model summary* |  |  |  |  |
| *R^2^* | 0.957 |  |  |  |
| *λ* | 0.429 |  |  |  |
| *Medulla oblongata ~ Rest of brain + Female group size (Rest of brain, Dimorphism)* | | | | |
|  | *b* | *se* | *t* | *p* |
| *Rest of brain* | 0.835 | 0.027 | 31.360 | <0.000 |
| *Female group size* | -0.066 | 0.030 | -2.214 | 0.032 |
| *Model summary* |  |  |  |  |
| *R^2^* | 0.978 |  |  |  |
| *λ* | 0.364 |  |  |  |
| ^1^Rest of brain is calculated as Total brain minus the dependent variable. ^2^Brain data were pooled from [3, 5]; predictors were pooled from [27, 35, 67]. | | | | |
