# Supplementary material for: Whatever you want: Inconsistent results are the rule, not the exception, in the study of primate brain evolution
Source: PLoS One. 2019 Jul 22;14(7):e0218655. doi: 10.1371/journal.pone.0218655 (PMC6645455; doi:10.1371/journal.pone.0218655)
Supplement: S11 Table — (DOCX) [file pone.0218655.s012.docx]

| Table S11. Data used to reevaluate Lindenfors (27) | | | | | | | | | | | | | |
| --- | --- | --- | --- | --- | --- | --- | --- | --- | --- | --- | --- | --- | --- |
| *Species* | *Male Weight (g)* | *Female Weight* | *Male group size* | *Female group size* | *Total brain* | *Telencephalon* | *Neocortex* | *Hippocampus* | *Striatum* | *Diencephalon* | *Cerebellum* | *Mesencephalon* | *Medulla oblongata* |
| *Aotus trivirgatus* | 813 | 834.5 | 1.1 | 1 | 16557.54 | 12609.92 | 9812.74 | 491.04 | 746.89 | 1110.05 | 1748.23 | 364.9 | 723.955 |
| *Ateles geoffroyi* | 7780 | 7449.5 | 4 | 17 | 101034 | 79946 | 70856 | 1366 | 4950 | 5334 | 12438 | 1482 | 1834 |
| *Avahi laniger* | 1030 | 1320 | 1 | 1 | 9798 | 6550 | 4813 | 526 | 524 | 862 | 1489 | 343 | 553 |
| *Callicebus moloch* | 1020 | 956 | 1 | 1 | 17944 | 13465 | 11163 | 588 | 920 | 1375 | 1622 | 530 | 787 |
| *Callimico goeldii* | 499 | 476.5 | 1 | 2 | 11311.17 | 8431.08 | 6333.72 | 250.79 | 483.75 | 808.36 | 1255.2 | 334.24 | 483.31 |
| *Callithrix jacchus* | 317 | 324 | 2.7 | 2.9 | 7682.88 | 5816.46 | 4456.49 | 212.1 | 353.13 | 537.73 | 721.62 | 257 | 350.58 |
| *Callithrix pygmaea* | 110 | 100.5 | 1.5 | 1 | 4305.17 | 3150.94 | 2388.06 | 107.24 | 167.3 | 340.49 | 473.06 | 176.61 | 164.075 |
| *Cebus apella* | 3650 | 2504.5 | 3.2 | 4.9 | 74229.03 | 61387.98 | 37879.4 | 548.09 | 2802.43 | 3784.61 | 5931.26 | 854 | 2271.17 |
| *Cercopithecus ascanius* | 3700 | 2910.5 | 1 | 8.5 | 63505 | 51279 | 45166 | 1189 | 2827 | 3605 | 5828 | 1162 | 1632 |
| *Cercopithecus mitis* | 7697 | 4495.5 | 1.1 | 8.8 | 68503.93 | 55411.05 | 42414.19 | 1074.49 | 2505.44 | 3844.69 | 6095.62 | 1133.32 | 2019.255 |
| *Cercopithecus nictitans* | 6670 | 4260 | 1 | 2 | 73183.47 | 59159.74 | 37132.31 | 806.42 | 2124.61 | 3367.65 | 7283.92 | 996.65 | 2375.51 |
| *Colobus guereza* | 13500 | 8353 | 1.3 | 2.9 | 77247.81 | 60744.88 | 33524.27 | 1127.59 | 2160.5 | 4331.59 | 7926.48 | 998.71 | 3246.14 |
| *Daubentonia madagascariensis* | 2620 | 2490 | 1 | 1 | 42611 | 30196 | 22127 | 1776 | 2765 | 3539 | 6461 | 897 | 1517 |
| *Erythrocebus patas* | 12400 | 5700 | 1.2 | 10.4 | 100086.4 | 83144.02 | 63082.54 | 1292.85 | 3026.93 | 4948.7 | 8018.01 | 1393.39 | 2582.79 |
| *Eulemur fulvus* | 2000 | 2205 | 3.2 | 3.5 | 22106 | 15566 | 12207 | 752 | 1215 | 1713 | 3328 | 590 | 909 |
| *Galago demidoff* | 63 | 64.5 | 1 | 1 | 3203 | 2240 | 1568 | 152 | 169 | 246 | 413 | 135 | 169 |
| *Galago senegalensis* | 227 | 193.5 | 1 | 1 | 4043.45 | 2669.35 | 1906.95 | 203.41 | 172.76 | 334.51 | 625.03 | 174.39 | 239.185 |
| *Gorilla gorilla gorilla* | 169367 | 77422 | 1.8 | 4.4 | 435339.2675 | 344267.15 | 255646.545 | 3232.23 | 11216.14 | 18025.6425 | 60733.805 | 3568.215 | 8743.955 |
| *Hylobates lar* | 5900 | 5360.5 | 1 | 1 | 97505 | 76001 | 65800 | 2673 | 4784 | 5716 | 12078 | 1459 | 2251 |
| *Indri indri* | 5830 | 6840 | 1 | 1 | 36285 | 25551 | 20114 | 1520 | 1855 | 2911 | 5504 | 978 | 1342 |
| *Lagothrix lagotricha* | 7280 | 7020 | 5.8 | 8.2 | 93589.85 | 74274.6 | 56371.03 | 1314.93 | 4147.41 | 5271.35 | 10487.78 | 1296.85 | 2259.28 |
| *Lepilemur ruficaudatus* | 761 | 779 | 1 | 1 | 7175 | 1708 | 3282 | 392 | 366 | 593 | 1165 | 260 | 449 |
| *Lophocebus albigena* | 8250 | 5998 | 4.8 | 7.8 | 93706.71 | 75484.63 | 56812.61 | 1277.83 | 3547.56 | 4850.32 | 9425.88 | 1350.7 | 2610.69 |
| *Loris tardigradus* | 192 | 193 | 1 | 1 | 6269 | 4551 | 3524 | 191 | 351 | 538 | 728 | 220 | 233 |
| *Macaca fascicularis* | 5360 | 3554 | 4.6 | 10.6 | 53845.39 | 43435.73 | 26848.31 | 534.01 | 1908.77 | 2851.41 | 5145.91 | 718.12 | 1694.23 |
| *Macaca fuscata* | 11000 | 8030 | 7.3 | 17.5 | 89294.87 | 75235.99 | 48310.08 | 850.76 | 3370.82 | 4396.61 | 6413.89 | 999.07 | 2249.31 |
| *Macaca mulatta* | 9355 | 6377.5 | 9.1 | 23.8 | 88121.58 | 71978.58 | 54019.95 | 1042.3 | 3421.89 | 4340.67 | 8321.27 | 1202.15 | 2279.405 |
| *Macaca nemestrina* | 9450 | 6119.5 | 2.4 | 14.9 | 94813.84 | 77846.96 | 42872.64 | 793.17 | 3148.98 | 5478.69 | 7126.3 | 1194.62 | 3167.27 |
| *Macaca silenus* | 8900 | 6100 | 3.1 | 7.1 | 95121.19 | 78735.7 | 51109.63 | 886.79 | 2515.04 | 4412.88 | 8282.98 | 1098.63 | 2591.01 |
| *Macaca sylvanus* | 16000 | 10000 | 5.2 | 7 | 83284.63 | 68216.51 | 45352.78 | 923.45 | 3096.24 | 4470.4 | 7259.82 | 1124.14 | 2213.75 |
| *Microcebus murinus* | 59 | 63 | 1 | 1 | 1688.15 | 1082.68 | 751.51 | 85.16 | 74.72 | 154.31 | 245.72 | 90.04 | 116.055 |
| *Miopithecus talapoin* | 1940 | 1560 | 13 | 21.3 | 37776 | 30166 | 26427 | 705 | 1908 | 2375 | 3374 | 826 | 1035 |
| *Nasalis larvatus* | 20400 | 9775 | 1 | 3.7 | 92797 | 70873 | 62685 | 1966 | 3735 | 5310 | 12113 | 1556 | 2945 |
| *Nycticebus coucang* | 679 | 588.5 | 1 | 1 | 11755 | 8495 | 6192 | 566 | 760 | 1077 | 1310 | 345 | 528 |
| *Otolemur crassicaudatus* | 1190 | 1018 | 1 | 1 | 9668 | 6545 | 4723 | 461 | 556 | 785 | 1414 | 384 | 540 |
| *Pan paniscus* | 45000 | 33200 | 7 | 8.9 | 306268.19 | 241237.58 | 143537.04 | 2283.81 | 7444.96 | 13072.52 | 42118.37 | 2031.04 | 8563.03 |
| *Pan troglodytes troglodytes* | 49567 | 40367 | 6.7 | 12.4 | 357703.89 | 285447.57 | 197764.97 | 2851.95 | 9719.14 | 15002.77 | 46818.36 | 2636.85 | 7798.59 |
| *Papio anubis* | 23150 | 13734.5 | 9.1 | 17.5 | 190957 | 154987 | 140142 | 3398 | 7182 | 9280 | 18683 | 2711 | 5297 |
| *Papio hamadryas* | 18950 | 10325 | 6.5 | 8.2 | 168266.3 | 139593.78 | 85309.2 | 1513.5 | 4566.84 | 7643.95 | 14639.32 | 1580.78 | 4808.47 |
| *Perodicticus potto* | 830 | 852 | 1 | 1 | 13212 | 9418 | 6683 | 607 | 712 | 1024 | 1699 | 391 | 680 |
| *Piliocolobus badius* | 8360 | 7671.5 | 4.4 | 11.4 | 73818 | 57885 | 50906 | 1671 | 3217 | 3945 | 8648 | 1333 | 2007 |
| *Pongo pygmaeus* | 78200 | 36324 | 1 | 1 | 323450.54 | 263834.03 | 164492.87 | 1628.69 | 7347.14 | 12184.13 | 37797.51 | 1994.97 | 7639.9 |
| *Propithecus verreauxi* | 3250 | 2950 | 3 | 2.8 | 25194 | 17040 | 13170 | 1043 | 1411 | 2195 | 3957 | 780 | 1223 |
| *Saguinus fuscicollis* | 343 | 385 | 1.5 | 1.5 | 8200.26 | 6176.23 | 4246.02 | 210.48 | 324.77 | 592.4 | 827.38 | 222.27 | 381.98 |
| *Saguinus oedipus* | 418 | 424.5 | 2.6 | 1.9 | 10294.52 | 7979.27 | 5962.29 | 229.59 | 449.26 | 722.17 | 910.95 | 283.8 | 398 |
| *Saimiri sciureus* | 899 | 722.5 | 2.7 | 7.9 | 22107.28 | 17696.3 | 13865.51 | 274.01 | 912.97 | 1310.58 | 1965.99 | 441.16 | 693.76 |
| *Varecia variegata variegata* | 3470 | 3255 | 2.5 | 3 | 29713 | 20461 | 15293 | 1404 | 1591 | 2529 | 4286 | 1018 | 1420 |
