# Supplementary material for: Whatever you want: Inconsistent results are the rule, not the exception, in the study of primate brain evolution
Source: PLoS One. 2019 Jul 22;14(7):e0218655. doi: 10.1371/journal.pone.0218655 (PMC6645455; doi:10.1371/journal.pone.0218655)
Supplement: S12 Table — (DOCX) [file pone.0218655.s013.docx]

| Table S12. Following Dunbar’s paper [19] that modelled the functional relationship between group size and neocortex ratio (neocortex to rest of the brain ratio) - this table shows three versions of this approach and the predicted group size for *Homo sapiens*. Read the table as follows: first, the fitted model on non-human primates, second, that model is used to predict group size of *Homo sapiens.* The first model uses only neocortex ratio as predictor with no control for phylogeny (as in Dunbar’s original paper), the second adds control for phylogeny and the third adds control for phylogeny and weight. | | | | |
| --- | --- | --- | --- | --- |
| Group size ~ Neocortex ratio (no control for phylogeny) | | | | |
|  | *b* | *se* | *t* | *p* |
| Neocortex ratio | 24.640 | 7.449 | 3.308 | 0.002 |
| Model summary |  |  |  |  |
| R^2^ | 0.159 |  |  |  |
| Using the above model to predict *Homo sapiens* group size: | | | | |
| -0.509 + 24.639 * *Homo sapiens* Neocortex Ratio = log(221.358) | | | | |
| Group size ~ Neocortex ratio (with control for phylogeny) | | | | |
|  | *b* | *se* | *t* | *p* |
| Neocortex ratio | 12.152 | 4.327 | 2.809 | 0.007 |
| Model summary |  |  |  |  |
| R^2^ | 0.159 |  |  |  |
| λ | 0.924 |  |  |  |
| Using the above model to predict *Homo sapiens* group size: | | | | |
| 0.185 (intercept) + 12.151 (*b)* * *Homo sapiens* Neocortex ratio = log(22.195) | | | | |
| Group size ~ Neocortex ratio + Weight (with control for phylogeny) | | | | |
|  | *b* | *se* | *t* | *p* |
| Neocortex ratio | 12.072 | 4.413 | 2.735 | 0.008 |
| Weight | 0.097 | 0.104 | 0.934 | 0.354 |
| Model summary |  |  |  |  |
| R^2^ | 0.343 |  |  |  |
| λ | 0.913 |  |  |  |
| Using the above model to predict *Homo sapiens* group size: | | | | |
| -0.519 (intercept) + 12.071 (*b_n_)* * *Homo sapiens* Neocortex Ratio + 0.097 (*b_w_)* * *Homo sapiens* Weight = log(31.242) | | | | |
