# Supplementary material for: Whatever you want: Inconsistent results are the rule, not the exception, in the study of primate brain evolution
Source: PLoS One. 2019 Jul 22;14(7):e0218655. doi: 10.1371/journal.pone.0218655 (PMC6645455; doi:10.1371/journal.pone.0218655)
Supplement: S13 Table — (DOCX) [file pone.0218655.s014.docx]

| Table S13. Data used to reevaluate Dunbar (19) | | | | |
| --- | --- | --- | --- | --- |
| *Species* | *Total brain* | *Neocortex* | *Weight* | *Group size* |
| *Aotus lemurinus* | 14646.570 | 7632.450 | 861.615 | 3.500 |
| *Aotus trivirgatus* | 16557.540 | 9812.740 | 847.275 | 3.706 |
| *Ateles fusciceps* | 104988.090 | 53484.990 | 8529.485 | 5.000 |
| *Ateles geoffroyi* | 101034.000 | 70856.000 | 7163.275 | 35.009 |
| *Avahi laniger* | 9798.000 | 4813.000 | 1237.500 | 2.833 |
| *Callicebus moloch* | 17944.000 | 11163.000 | 935.903 | 3.633 |
| *Callimico goeldii* | 11311.170 | 6333.720 | 513.125 | 6.250 |
| *Callithrix geoffroyi* | 7098.630 | 4057.730 | 350.500 | 9.000 |
| *Callithrix jacchus* | 7682.880 | 4456.490 | 296.603 | 8.139 |
| *Callithrix penicillata* | 6335.880 | 3264.950 | 325.120 | 6.950 |
| *Callithrix pygmaea* | 4305.170 | 2388.060 | 115.532 | 5.964 |
| *Cebus apella* | 74229.030 | 37879.400 | 2873.230 | 13.980 |
| *Cercopithecus ascanius* | 63505.000 | 45166.000 | 3730.998 | 25.147 |
| *Cercopithecus mitis* | 68503.930 | 42414.190 | 6215.840 | 22.288 |
| *Cercopithecus nictitans* | 73183.470 | 37132.310 | 5321.568 | 15.625 |
| *Cheirogaleus major* | 6373.000 | 2938.000 | 415.170 | 5.500 |
| *Cheirogaleus medius* | 2961.000 | 1221.000 | 235.690 | 3.750 |
| *Colobus guereza* | 77247.810 | 33524.270 | 10489.438 | 8.400 |
| *Daubentonia madagascariensis* | 42611.000 | 22127.000 | 2579.062 | 2.375 |
| *Erythrocebus patas* | 100086.400 | 63082.540 | 8454.075 | 27.360 |
| *Eulemur fulvus fulvus* | 22106.000 | 12207.000 | 2217.415 | 9.490 |
| *Galago senegalensis* | 4043.450 | 1906.950 | 210.331 | 1.000 |
| *Galagoides demidoff* | 3203.000 | 1568.000 | 62.993 | 2.625 |
| *Gorilla gorilla gorilla* | 435339.268 | 255646.545 | 122360.748 | 12.874 |
| *Hylobates lar* | 97505.000 | 65800.000 | 5546.560 | 3.757 |
| *Indri indri* | 36285.000 | 20114.000 | 8417.500 | 3.890 |
| *Lagothrix lagotricha* | 93589.850 | 56371.030 | 7398.948 | 21.055 |
| *Lepilemur ruficaudatus* | 7175.000 | 3282.000 | 770.000 | 1.000 |
| *Lophocebus albigena* | 93706.710 | 56812.610 | 7528.118 | 18.640 |
| *Loris tardigradus* | 6269.000 | 3524.000 | 231.153 | 1.500 |
| *Macaca arctoides* | 90976.420 | 47218.070 | 9101.965 | 13.750 |
| *Macaca fascicularis* | 53845.390 | 26848.310 | 4550.553 | 29.411 |
| *Macaca fuscata* | 89294.870 | 48310.080 | 9506.940 | 60.075 |
| *Macaca mulatta* | 88121.580 | 54019.950 | 7168.990 | 52.431 |
| *Macaca nemestrina* | 94813.840 | 42872.640 | 7514.672 | 38.430 |
| *Macaca nigra* | 75427.170 | 34402.600 | 7671.098 | 41.000 |
| *Macaca silenus* | 95121.190 | 51109.630 | 7217.563 | 18.300 |
| *Macaca sylvanus* | 83284.630 | 45352.780 | 12086.633 | 25.530 |
| *Mandrillus sphinx* | 128292.080 | 66858.270 | 21046.265 | 54.450 |
| *Microcebus murinus* | 1688.150 | 751.510 | 62.467 | 3.050 |
| *Miopithecus talapoin* | 37776.000 | 26427.000 | 1493.477 | 88.838 |
| *Nasalis larvatus* | 92797.000 | 62685.000 | 14264.663 | 9.470 |
| *Nycticebus coucang* | 11755.000 | 6192.000 | 736.050 | 1.000 |
| *Otolemur crassicaudatus* | 9668.000 | 4723.000 | 1235.602 | 2.625 |
| *Pan paniscus* | 306268.190 | 143537.040 | 38311.238 | 46.750 |
| *Pan troglodytes troglodytes* | 357703.890 | 197764.970 | 46647.500 | 38.854 |
| *Papio anubis* | 190957.000 | 140142.000 | 17825.885 | 52.323 |
| *Papio hamadryas* | 168266.300 | 85309.200 | 13319.930 | 36.313 |
| *Perodicticus potto* | 13212.000 | 6683.000 | 982.746 | 2.125 |
| *Piliocolobus badius* | 73818.000 | 50906.000 | 8060.375 | 30.240 |
| *Pongo pygmaeus* | 323450.540 | 164492.870 | 56270.573 | 2.300 |
| *Propithecus verreauxi* | 25194.000 | 13170.000 | 3244.710 | 6.710 |
| *Pygathrix nemaeus* | 72530.000 | 48763.000 | 9485.183 | 15.375 |
| *Saguinus fuscicollis* | 8200.260 | 4246.020 | 385.415 | 5.350 |
| *Saguinus imperator* | 9370.880 | 5012.380 | 474.500 | 6.000 |
| *Saguinus midas* | 10175.940 | 5873.080 | 530.102 | 5.700 |
| *Saguinus oedipus* | 10294.520 | 5962.290 | 405.048 | 6.099 |
| *Saimiri boliviensis* | 21657.870 | 12418.810 | 759.113 | 40.000 |
| *Saimiri sciureus* | 22107.280 | 13865.510 | 811.120 | 33.654 |
| *Varecia variegata variegata* | 29713.000 | 15293.000 | 3348.750 | 6.217 |
